# Global Climate Implications for Homelessness: A Scoping Review

**DOI:** 10.1007/s11524-020-00483-1

**Published:** 2020-09-23

**Authors:** Sean A. Kidd, Susan Greco, Kwame McKenzie

**Affiliations:** 1 Department of Psychiatry, University of Toronto, Toronto, Canada; 2 Division Chief – Psychology, Centre for Addiction and Mental Health, 1001 Queen St. W., Unit 2-1, #161, Toronto, Ontario M6J 1H4 Canada; 3 Dalla Lana School of Public Health, University of Toronto, Toronto, Canada

**Keywords:** Homelessness, Climate, Review, Systems

## Abstract

Homelessness is a persistent global challenge with significant health impacts on those affected. Homeless people are by definition the most exposed to weather conditions and the social and economic problems caused by extreme weather and climate change and variability. This systematic review was designed to synthesize the academic literature that addresses the health and social implications of global climate change for homelessness. The question examined in this systematic scoping review is the following: What is the current state of knowledge in the scientific literature on the health and social implications of global climate change for homelessness? A systematic scoping review method was used to identify and synthesize the peer-reviewed literature relevant to this question. The databases searched were PsycINFO, Medline, Scopus, and Google Scholar. Of the 26 papers identified in this review, 20 employed original data analyses with conclusions largely inferred from cross-sectional associations. Themes included the potential influence of climate change on homelessness prevalence, climate impacts that exacerbate specific vulnerabilities of homeless populations (e.g., chronic illness, exposure, stigmatization), and health and social outcomes. Service use and design implications were also addressed. Given the scale of the impacts of climate change on homelessness, the literature on this topic poses promising directions but is under-developed in its current state to adequately inform risk mitigation and response planning. A systems framework is proposed here to inform future research and service design.

Global climate change has been described as “the defining issue of our time” [44]. Climate refers to weather parameters such as temperature, rainfall, and wind speed that occur in a given place over a given period of time [46]. However, such a general definition belies the complexity of the phenomenon and the dangers that it poses [12]. To date, large volumes of evidence describe and predict the potential adverse effects of climate change [7]. In this literature, human health and social impacts figure prominently. Extremes of temperature, coupled with humidity levels, significantly impact mortality rates as do extreme weather events [18, 21]. Temperature and humidity also influence illness morbidity (e.g., respiratory and cardiovascular conditions) [17], and have implications for the progression of vector-borne diseases [33], with differential risks as a function of age with risk increasing at the youngest and oldest ends of the spectrum [3, 11]. The social impacts of climate extremes and changes include pronounced impacts on rates of interpersonal and intergroup violence [22], social and health infrastructure [8], and migration [4]. A cross-cutting theme of these various interactions is inequality—with disproportionately larger effects on the lives of the impoverished and stigmatized who also increase in number due to climate impacts. Gender figures prominently with climate effects disproportionately higher for girls and women in all domains (e.g., mortality, HIV infection, education) [7].

Homelessness, in turn, is a persistent global challenge with major health impacts on those affected. It is estimated that over 100 million people are homeless globally, with 1 billion living in precarious housing arrangements [43]. Homeless people are by definition the most exposed to weather conditions and the social and economic problems caused by extreme weather and climate change. The umbrella term “homelessness” belies a dimensional construct [14] ranging from being roofless and sleeping rough, being houseless in shelter environments, through to various forms of insecure and inadequate housing—all of which have unique implications as they relate to climate change and weather exposure. Thus, it is important to consider the possible consequences of climate change for homeless populations. This topic has, to date, received relatively little attention and preparedness for the impacts of a changing climate on homelessness populations is in question. Accordingly, this systematic review was designed to synthesize the academic literature that addresses the health and social implications of global climate change for homelessness.

## Methods

Scoping review methods are designed to identify and articulate concepts and evidence in areas where the topic of investigation is complex and (or) when the topic is reviewed for the first time. Global climate change is complex in terms of types of impact, systems involved, and the associated fields of study. Furthermore, we did not identify a previous systematic review that addresses this topic. As such, we have followed the Arksey and O’Malley [2] five-stage scoping review framework of (1) identifying the research question, (2) identifying relevant results, (3) selecting studies, (4) charting data, and (5) reporting results. Our description of the scoping review design was further informed by Levac et al. [29]. The authors of the present review have expertise in homelessness (SK), population health and marginality (KM), and the public health implications of weather and climate change (SG).

First, the question examined in this review is as follows: What is the current state of knowledge in the scientific literature on the health and social implications of global climate change for homelessness? The breadth of this question was necessary due to the early stage of the literature on this topic. Second, an a priori search strategy was developed to identify the peer-reviewed literature relevant to this question. The search, conducted in November 2019, covered the period from inception to November 2019, and was restricted to English language peer-reviewed journals. The databases searched were PsycINFO, Medline, Scopus, and Google Scholar. The resulting search terms included for group one: “homeless*” and “climate”, “weather”, or “environment*”. Keywords were searched using “OR” within groups and “AND” in two separate searches to combine homelessness and keywords relevant to the climate change phenomenon. For Google Scholar, the search proceeded until 100 unsuccessful hits occurred after the last successful hit in the identified documents. In stage 3, we examined abstracts to ensure that they focused on climate and homelessness. We excluded papers at the abstract review level that did not address this combined topic and removed duplicates. We included papers that involved data collection and papers that did not (e.g., commentaries) given the early stage of the literature in this area and expert commentaries’ potential value. We also included papers that did not overtly address global climate change as the implications of those papers would seem relevant. A full-text review was completed of all articles selected through the abstract review (extracted by SK), with some further removal of papers that were out of scope and some additional identification of papers from reference lists. We charted [2, 38] the selected papers based upon (i) basic descriptors (e.g., year and country of publication, publication type, and method), (ii) a summary of the findings of studies involving data, and (iii) a thematic synthesis [30] of both research and commentary jointly interpreting the thematic analysis framework which was developed using the original text from the identified papers. Findings were summarized as a function of both coverage of key themes and methods used with a consensus process determining the research, policy, and practice implications of the findings. The consultation phase [29] of this program of work, of which the present review is the first step, will take place in a subsequent stage. That phase will involve the use of a Delphi method [23] to engage international experts in the field who will develop policy guidance based on their experience, the present review, and a review of reviews (underway) examining the climate change–related housing implications for impoverished populations.

## Results

**Fig. 1:**
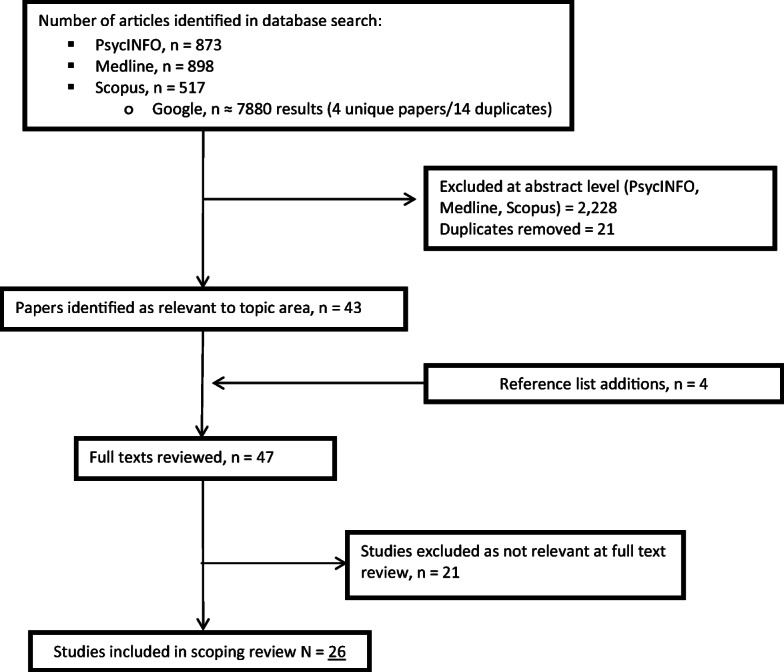
Article selection flow diagram

**Fig. 2:**
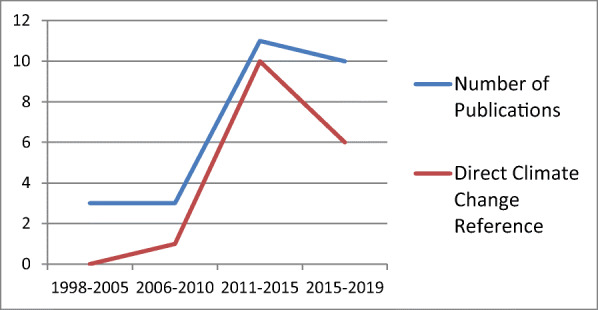
Histogram of climate change and homelessness publications (1998–2019)

The search results are detailed in Fig. 1. PsycINFO generated 1420 results, which, in turn, were reduced to 873 when limited to English language and peer-reviewed journals. Medline yielded 942 results that were reduced to 898 when limited to English. Scopus generated 517 results, and Google Scholar generated 7880 results. Of the papers identified in the search, 43 articles were selected as meeting the search criteria after removing duplicates (*n* = 21). Additional 4 articles were identified from reference lists. The full texts of these 47 papers were then reviewed, with 21 subsequently removed as not meeting criteria, leaving 26 for which their content was charted (Table 1). The 26 included publications spanned the years 1998 to 2019 (Fig. 2). The majority were authored in the USA (*n* = 13), followed by Australia (*n* = 4) and Canada (*n* = 4), with single papers from Poland, Nigeria, India, the UK, and Venezuela. Twenty-one papers involved data collection with the remainder consisting of commentaries and narrative reviews (none systematic). Of the 21 research papers, 15 were quantitative, 5 were qualitative, and one used mixed methods.

**Table 1:** Article summary

Paper	Country
Hale [19]. Fountains for environmental justice: public water, homelessness, and migration in the face of global environmental change.	USA
Zhang et al. [48]. Cold weather conditions and risk of hypothermia among people experiencing homelessness: implications for prevention strategies.	Canada
*Gibson [16]. Climate change for individuals experiencing homelessness: recommendations for improving policy, research, and services.	USA
Every et al. [13]. There’s nowhere to go: counting the costs of extreme weather to the homeless community.	Australia
Corinth and Lucas [9]. When warm and cold do not mix: the implications of climate for the determinants of homelessness.	USA
Romaszko et al. [39]. Mortality among the homeless: causes and meteorological relationships.	Poland
Settembrino [40]. “Sometimes You Cannot Even Sleep at Night:” social vulnerability to disasters among men experiencing homelessness in Central Florida.	USA
Nicolay et al. [32]. A study of heat related illness preparedness in homeless veterans.	USA
Quilty et al. [36]. Factors contributing to frequent attendance to the emergency department of a remote Northern Territory hospital.	Australia
Lee et al. [27]. Geographic distribution of disaster-specific emergency department use after Hurricane Sandy in New York City.	USA
Adetokunbo and Emeka [1]. Urbanization, housing, homelessness and climate change.	Nigeria
*Campbell [6]. Let us not forget climate change in the food insecurity conversation: why the homeless are most vulnerable.	Australia
Walters and Gaillard [45]. Disaster risk at the margins: homelessness, vulnerability and hazards.	India
Siordia et al. [42]. A geographically-aware multilevel analysis on the association between atmospheric temperature and the “Emergency and transitional shelter population”.	USA
Pepper and Jocoy [35]. A climatological analysis of emergency homeless shelter openings in Long Beach, California, USA.	USA
Cusack et al. [10]. Extreme weather-related health needs of people who are homeless.	Australia
Harlan et al. [20]. Neighborhood effects on heat deaths: social and environmental predictors of vulnerability in Maricopa County, Arizona.	USA
*Parlee and Furgal [34]. Well-being and environmental change in the arctic: a synthesis of selected research from Canada’s International Polar Year program.	Canada
*Shonkoff et al. [41]. The climate gap: environmental health and equity implications of climate change and mitigation policies in California—a review of the literature.	USA
Klein and Riemer [24]. Experiences of environmental justice and injustice in communities of people experiencing homelessness.	Canada
Brown et al. [5]. Do emergency department attendances by homeless people increase in cold weather?.	UK
Kloos et al. [25]. Investigating the roles of neighborhood environments and housing-based social support in the relocation of persons made homeless by hurricane Katrina.	USA
*Ramin and Svoboda [37]. Health of the homeless and climate change.	USA
Koutsavlis and Kosatsky [26]. Environmental-temperature injury in a Canadian metropolis.	Canada
Wiesenfeld and Panza [47]. Environmental hazards and home loss: the social construction of becoming homeless.	Venezuela
North et al. [31]. The association of psychiatric diagnosis with weather conditions in a large urban homeless sample.	USA

*Primarily review and/or commentary

Number of articles identified in database search: PsycINFO, *n* = 873; Medline, *n* = 898; Scopus, *n* = 517; Google, *n* ≈ 7880 results (4 unique papers/14 duplicates)

None of the identified papers provided causal data that links global climate change with a change in homelessness prevalence, morbidity, or mortality. The effects of climate change were typically inferred from observations of the associations between weather and the experiences and outcomes of homeless persons. The degree to which climate change was addressed varied. Some papers concentrated solely on the effects of weather extremes upon homelessness and change over time, without overtly addressing global climate change [47]. The earliest paper that described climate change and homelessness directly was authored by Ramin and Svoboda in 2009. The proportions of papers that directly addressed climate change versus weather impacts alone are shown in Fig. 2. The impacts of temperature extremes and climate change on homelessness were viewed almost without exception as negative. Only one paper made some conjectures about the possible benefits of climate change for some homeless populations (e.g., those residing in Canada). Ramin and Svoboda [37] noted that in generally colder climates, warmer weather may lead to fewer deaths from cold exposure and illnesses such as influenza, with the caveat that risks may outweigh these benefits. There was also considerable variability across papers regarding how the construct of homelessness was defined. Five papers, primarily commentaries, provided no clear definition and used the term broadly, and 7 papers relied upon a homeless categorization derived from electronic medical records or administrative data sets with little detail on definition aside from the indication of no fixed abode or similar. Six papers concentrated on shelter-using populations, with service use status as the indicator of homelessness, and 5 papers described their populations as having experienced “absolute” or “primary” homelessness with some variability as how this categorization addressed (i.e., sleeping rough (roofless) or some emergency shelter use). These latter papers tended to address the dimensionality of homelessness to a greater degree (e.g., [45]), with Every et al. [13] applying the European Typology of Homelessness and Housing Exclusion definition of degrees of homelessness and housing precarity. The most nuanced commentary on homelessness as dimensional and intertwined with social inequities described these phenomena in low income countries where it was argued that housing precarity and homelessness must be considered differently than the situations in high income countries [1, 47].

This review is organized into four themes as they emerged in the thematic synthesis. First is the topic of weather and climate change as a driver of homelessness prevalence. The second theme addresses how the conditions of homelessness serve to increase vulnerability to weather extremes. The third theme addresses the role of extreme weather and variability in the health of homeless populations. The fourth theme addresses service access and design.

### Weather and Climate Change and the Prevalence of Homelessness

This literature provided little data-derived information about how weather and climate change may serve as a driver of increasing homeless numbers. This topic was generally addressed with broad statements about large-scale risks inferred from climate data [16], with some emphasizing unique vulnerabilities faced in low-income countries (e.g., “The phenomenon of climate change heralds distinctive challenges for sub-Saharan Africa’s urban areas, with economic, social, and health impacts, and severe effects on housing and infrastructure.” [1], p.15; [47]). An exception was Every et al. [13], wherein a survey of homeless sector service providers in Australia indicated an estimate that extreme weather was a pathway to homelessness for 16.2% of service users.

### Vulnerability and Exposure

The topic of vulnerability was addressed primarily through commentary that emphasized the weather exposure risks (both temperature and extreme weather events such as flooding) that are greatly heightened for the homeless [16, 41, 45]. This theme was highlighted across settings internationally, including the Arctic and icecap melting [34]. A case study of Lagos, Nigeria, highlighted the flood and broader weather exposure risks for slum dwellers and squatters that result from both the quality and location of their dwellings [1], with similar themes addressed by Wiesenfeld and Panza [47] in Venezuela regarding vulnerability to landslides. Papers addressing low-income contexts concentrated on severe poverty and the challenges of very marginally housed individuals who are more exposed and less able to recover from extreme weather events. While most of this literature concentrated on physical exposure, qualitative US research emphasized that exposure is an intersectional phenomenon that includes not just greater exposure to the elements but also intersects with factors such as social stigma and unemployment [40].

Chronic health conditions common to homeless populations create unique and increased vulnerability to conditions such as heat stroke, dehydration, and respiratory illness [13]. Malnourishment was referenced as a problem that reduced the capacity to tolerate cold exposure [45]. Also, the risk of death from heat is increased for those with psychiatric disabilities, alcoholism, and cognitive impairment—inferring risk from the high prevalence of these conditions among the homeless [37].

Access to means for mobility and social resources were also highlighted as exacerbating exposure to weather extremes. Authors commented on homeless persons’ limited ability to escape extreme weather and draw upon social resources in emergencies [13]. Social isolation, common among the homeless, was also flagged as a risk factor for death from extreme heat [37] as was public access to water which is increasingly privatized in urban environments [19]. Finally, some commentary addressed the impacts of climate change on food security, with the homeless being particularly vulnerable [6].

Some commentaries placed this increased exposure to climate-related risks among the homeless in the context of the “environmental injustices” experienced by this population [16]. These authors highlight the systemic inequities and discrimination that homeless people face which, in turn, both increase exposure to environmental risks and decrease access to assistance for the negative outcomes of those risks [16, 19]. However, homeless people themselves may be less likely to hold this perspective [24].

### Climate Change, Weather, and Health Outcomes

The health outcome risks that were associated with weather events were the area with the most quantitative data. In both Canada [26, 48] and Poland [39], heightened cold-related morbidity and mortality were observed for homeless populations, and both moderately cold and extremely cold temperatures present significant risks. Zhang et al. [48] also demonstrated health outcome risks related to increases in precipitation. Harlan et al. [20] described heat-related mortality in Arizona, noting that the mortality risk for homeless persons was particularly concentrated in the inner core and industrial corridor areas. They wrote of a “heat island” effect in which higher temperatures are observed in places where a large majority of homeless people seek shelter [37]. Another US study in this area described homeless individuals’ experiences, noted minimal recognition of the symptoms of heat-related illness aside from dizziness [32]. In an Australian study of homeless service provider impressions about the impacts of extreme weather [13], providers saw extreme weather events as negatively affecting the physical health of 18% of their clients, the mental health of 37%, and the drug and alcohol consumption of 26%. This study’s qualitative suggested that weather-related stressors such as the restriction of movement and disrupted social connections due to loss and evacuation create and compound mental health challenges. These observations were reflected in another qualitative study done in Australia in which the most physical and mental suffering was ascribed to extremely cold and wet weather—mainly due to wet bedding, clothing, and shoes [10]. Similarly, a US study observed greater rates of diagnoses of mental illnesses and addictions among homeless men when diagnostic interviews took place on days with cold and wet weather in St. Louis [31].

Finally, one study of Australian service provider perspectives suggested that interpersonal violence, as a key health determinant, is strongly affected by extreme weather for homeless populations. In this survey, providers attributed interpersonal violence and domestic violence to extreme weather at rates of 26.5% and 18.5%, respectively, suggesting that this increase was likely due to people unexpectedly being placed in close quarters for extended periods [13]. The only additional point in this area was that of Ramin and Svoboda [37]. They suggested the increased risk of water- and vector-borne illness for more exposed homeless populations in reference to both rising sea levels and extreme weather events.

### Services and Service Access

With respect to weather and service use, an Australian study suggested a modest relationship between frequent emergency service use by homeless persons in hotter, wetter weather [36], with a UK study finding no difference in general emergency department use by homeless persons as a function of cold weather [5]. Another US study noted a greater likelihood of emergency department use by homeless persons in weather emergencies such as hurricanes [27]. It was also suggested that transience and many homeless persons’ hidden locations hamper emergency response workers efforts in climate emergencies [13]. This may be a problem compounded by a lack of environmental emergency planning related to homeless populations [45].

Quantitative research examined ambient temperature as it related to emergency shelter availability. First, across the USA, it was observed that the numbers/concentrations of emergency and temporary shelter users were positively associated with warmer climate regions [42]. Another study examined the periods of emergency shelter operation in California against days with low temperature or rain and concluded that periods of operation adequately aligned with weather conditions, though noted that providers seemed primarily concerned with extreme heat relative to cold [35]. While research into the outcomes of interventions in this area was lacking, several authors made recommendations for service and system improvements. Suggestions included the education of homeless populations about the symptoms of heat-related illness [32], improved access to public water fountains [19], land use zoning and water management strategies to reduce exposure [1], and developing improved host strategies for individuals made homeless by natural disasters [25]. Finally, there was some commentary on how underfunding and poor planning adversely impacts the response to climate-exposed homeless populations in both an ongoing way and during disasters [13, 16].

### Tentative Model for Considering Climate-Homelessness Interrelationships

**Fig. 3:**
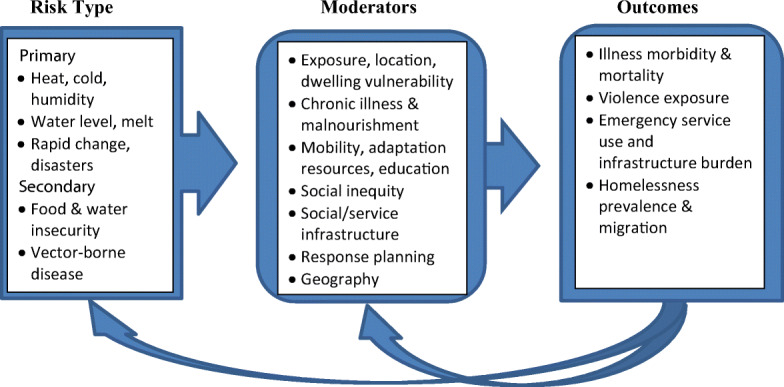
Hypothetical model for considering climate-homelessness relationships

Overall, this literature has several shortcomings, particularly due to the lack of data that might address causal relationships between homelessness, weather impacts, and climate change. However, there would seem to be enough information at hand, even if purely hypothetical at this stage, to posit a model that might capture the most substantive variables and their respective influences (Fig. 3). In this model the primary (e.g., increasing temperature and temperature variability) and secondary (e.g., water insecurity) impacts of climate change are moderated by variables that are specific to vulnerably housed and homeless populations from individual to system levels (e.g., chronic respiratory illness, malnutrition, location vulnerability, service planning, response, and infrastructure). The interactions between weather-related risks and variables that characterize homeless populations will determine outcomes at individual (e.g., mortality, violence exposure), system (e.g., emergency service use), and population levels (e.g., homelessness prevalence and migration). This model is supported in the literature reviewed, wherein the possibility of variable impacts as a function of specific climate stressors and moderators such as chronic illness on health and social outcomes was documented [13, 39]. What is attempted here, and what seemed to have yet to happen in the modest literature to date, is to organize these posited variables and relationships into a single framework. Much more hypothetical are possible feedback loops such as how outcomes might affect moderators over time from individual to population levels and, potentially, how outcomes such as mass homelessness and migration might themselves impact climate change.

## Discussion

This systematic scoping review found that the literature addressing the health and social impacts of weather and climate change on homelessness is limited to a small number of commentaries and analyses of primarily local, cross-sectional data. Nonetheless, it can be inferred from the small amount of data generated to date and the consideration of the nature of climate change, weather, and homelessness as conditions that homeless populations face unique vulnerabilities. These vulnerabilities likely lead to serious mental and physical health consequences. Furthermore, the prevalence of homelessness globally may be increasing due to the climate and weather vulnerabilities of marginally housed people. Based on this review, we have proposed a model that might capture this phenomenon (Fig. 3). While much more data would be necessary to validate this model, it is well-aligned with climate risk modeling initiatives underway that are grappling with multiple interactions and dynamic change from geographic to social levels over time [15].

The generation of risk mitigation and response strategies could, in turn, be developed and optimized with more robust risk modeling data. It is conceivable that a risk algorithm could help service systems and geographic regions develop multilevel and multistage approaches. For example, a given service system might become able to employ, with better evidence, more effective prevention, and risk mitigation strategies for specific homeless subpopulations as it relates to particular weather risks. Systems might also become better able to prepare for increases in numbers of homeless people due to extreme weather events. Prevention efforts might also be informed by feedback loops. Hypothetical examples include increased exposure to violence leading to an avoidance of services and the exacerbation of weather-related problems such as vector-borne disease. With some exceptions [16], the literature to date has not substantively explored systematic responses—responses that are beginning to emerge globally in the broader frame such as the Resilient Cities Program [28]. However, there are promising initial steps addressed in this review that might become a part of a systemic response. Examples in the literature reviewed include outreach and education for homeless and vulnerable populations [32], provider education [10], service adaptations and disaster planning [16]. Such strategies might be further developed using a model such as the one proposed in the present review. The model could inform how best to combine and sequence responses as a function of stage (current state–projected future states, risk mitigation, and prevention-crisis response) with contingencies to attend the dynamic and complex nature of climate impacts. More generally, as a function of homeless people as a highly stigmatized group, this review has highlighted the perspective that climate impacts on homelessness may exacerbate systematic and historical inequities along intersecting socioeconomic, racial, and cultural lines [7, 16, 34].

In conclusion, this scoping review has highlighted a potential model for considering the health and social risks that global climate change pose for homeless populations. The review must be regarded as preliminary, given the ranging foci and methods described in the identified papers. There is also ambiguity in how degrees of homelessness versus highly vulnerable housing are considered and the influences of geographic context, with relatively little coverage of phenomena relevant to low and middle-income countries where weather vulnerabilities are the most acute. From research to the design of service and system responses, clear articulation of degrees of homelessness/housing vulnerability (e.g., [14]) and the systemic inequities that they represent will be essential. Nonetheless, the themes highlighted in this review might serve to guide future studies that can address some of the knowledge gaps that reduce our collective ability to respond to this global problem.
